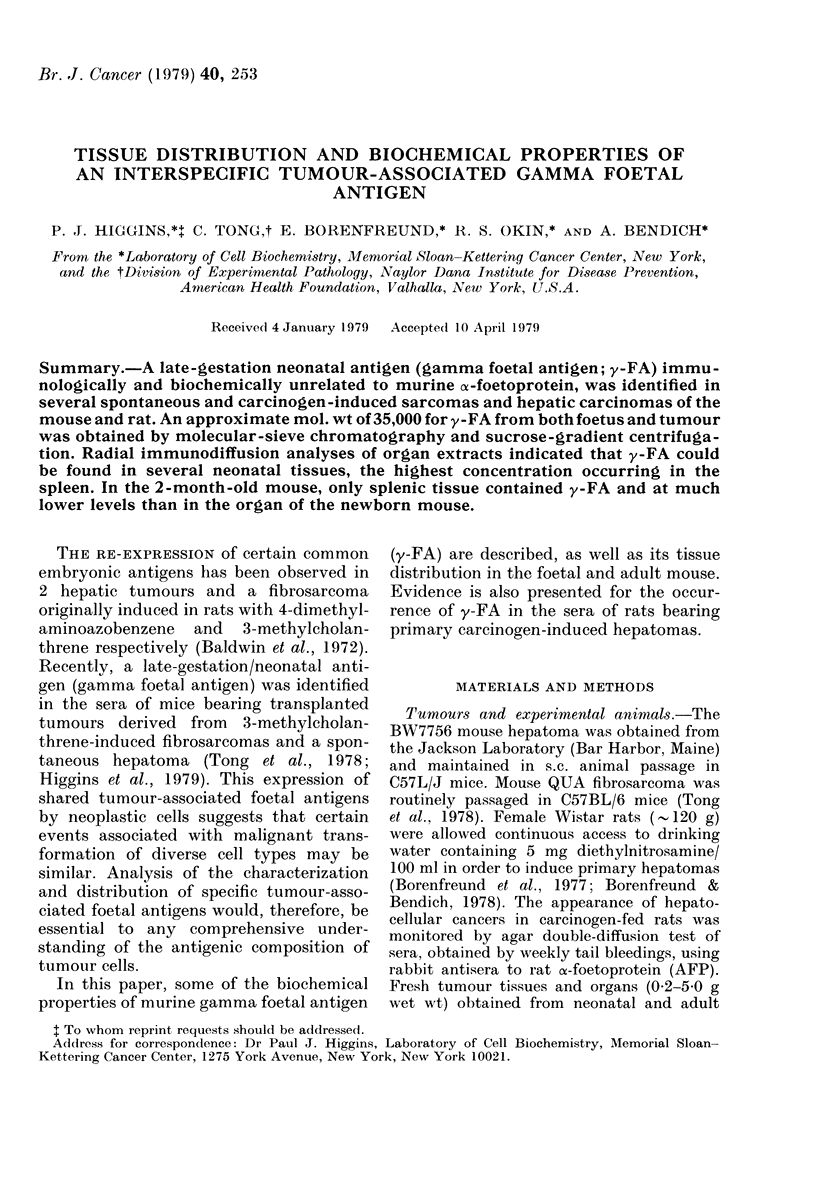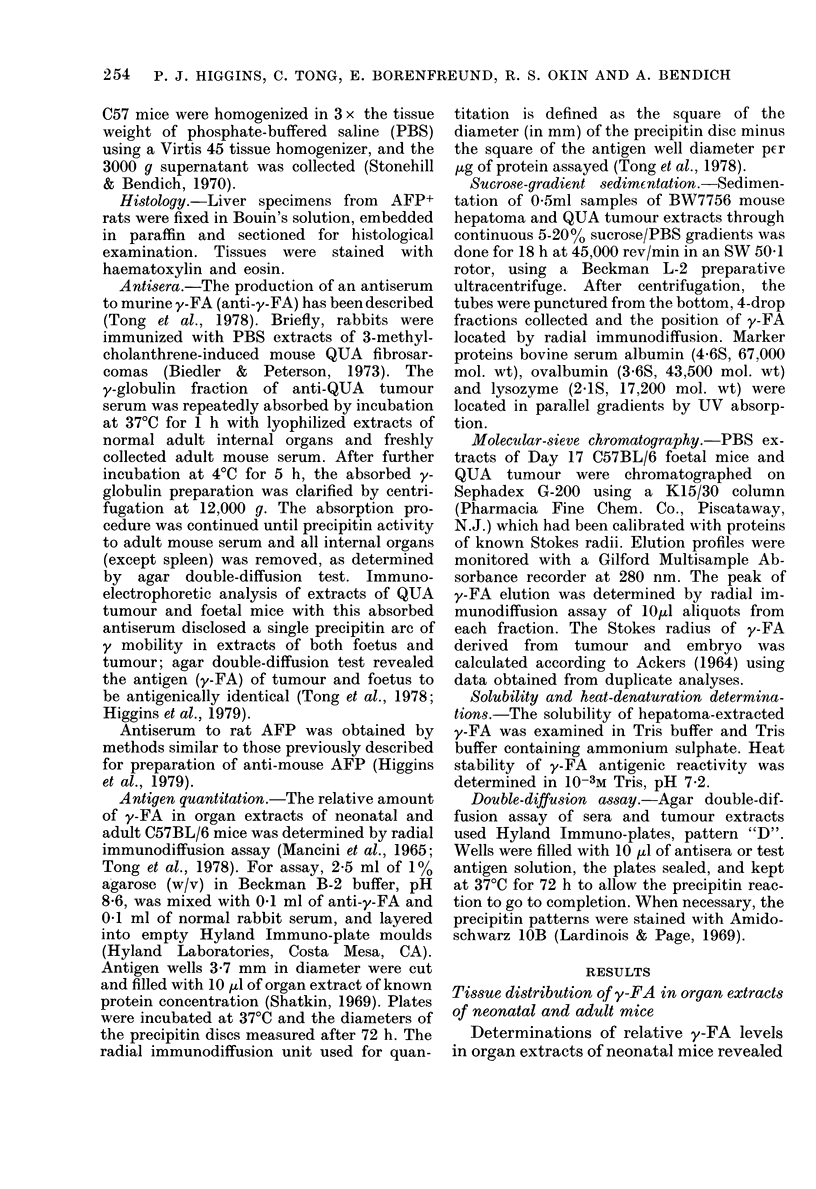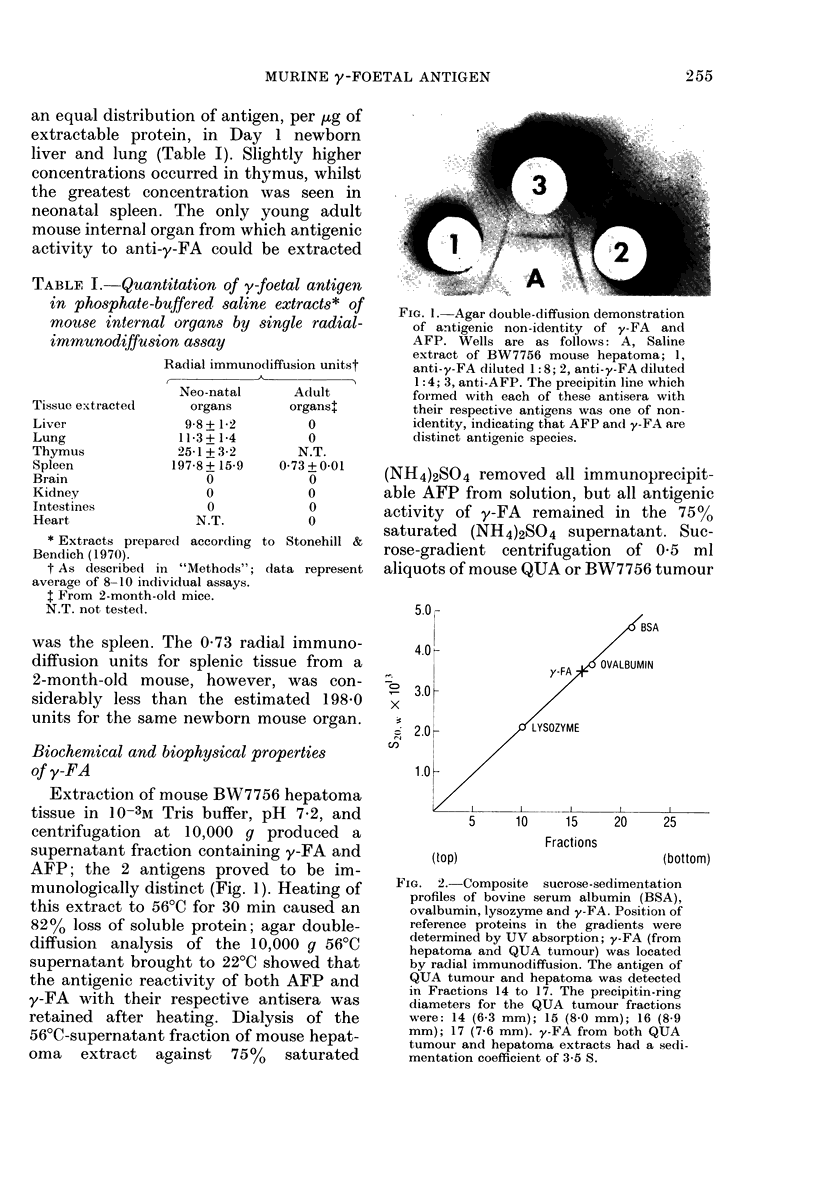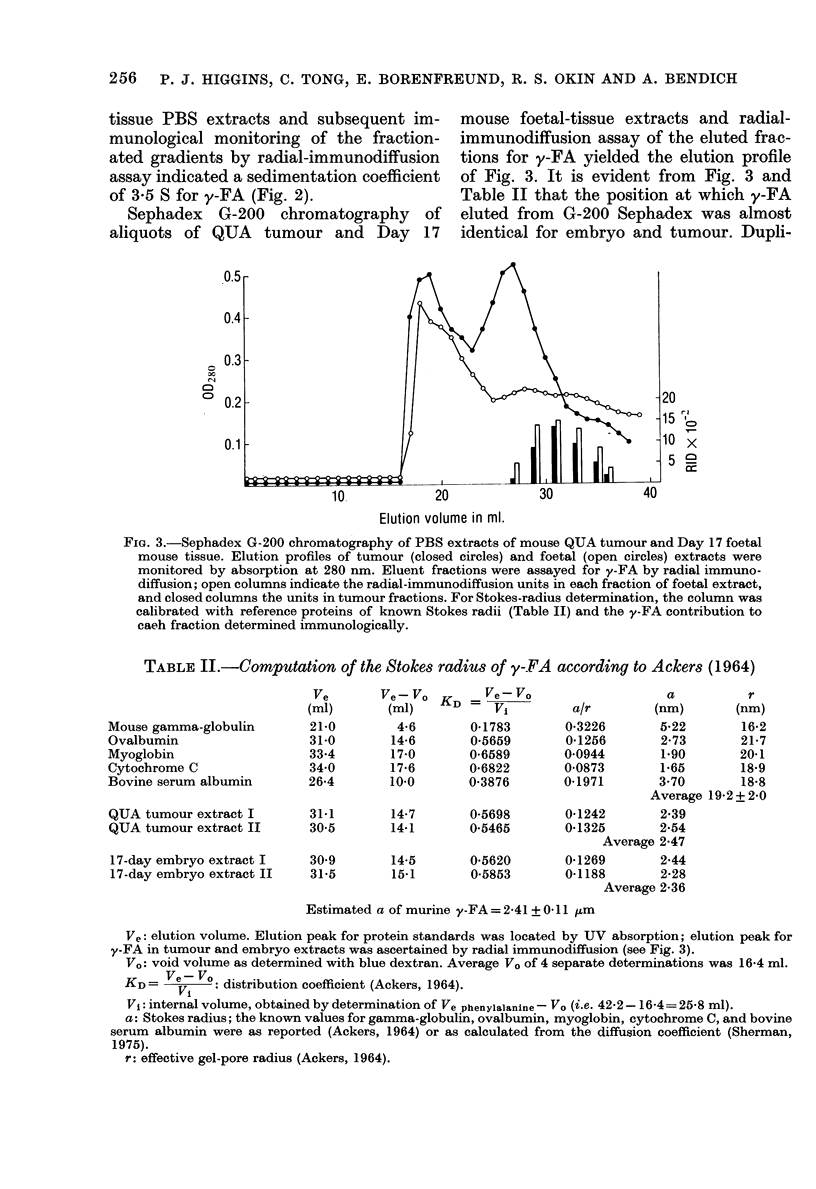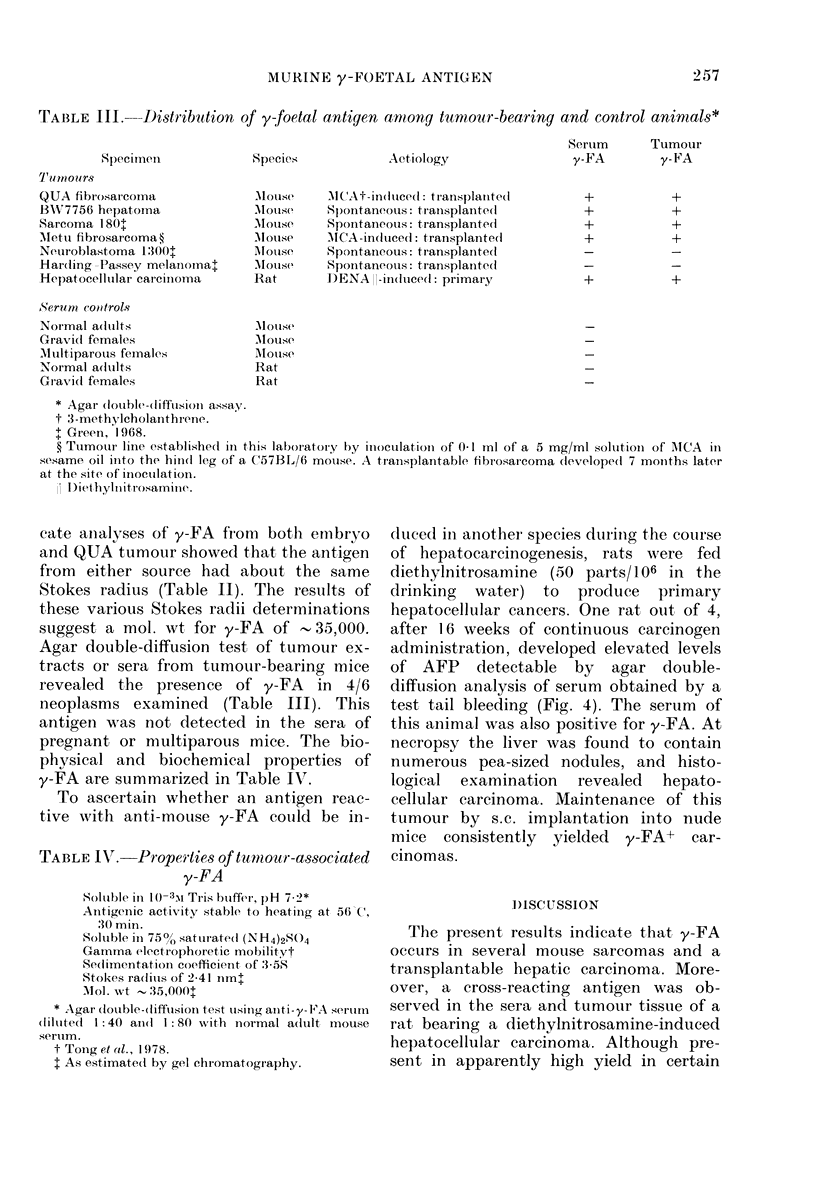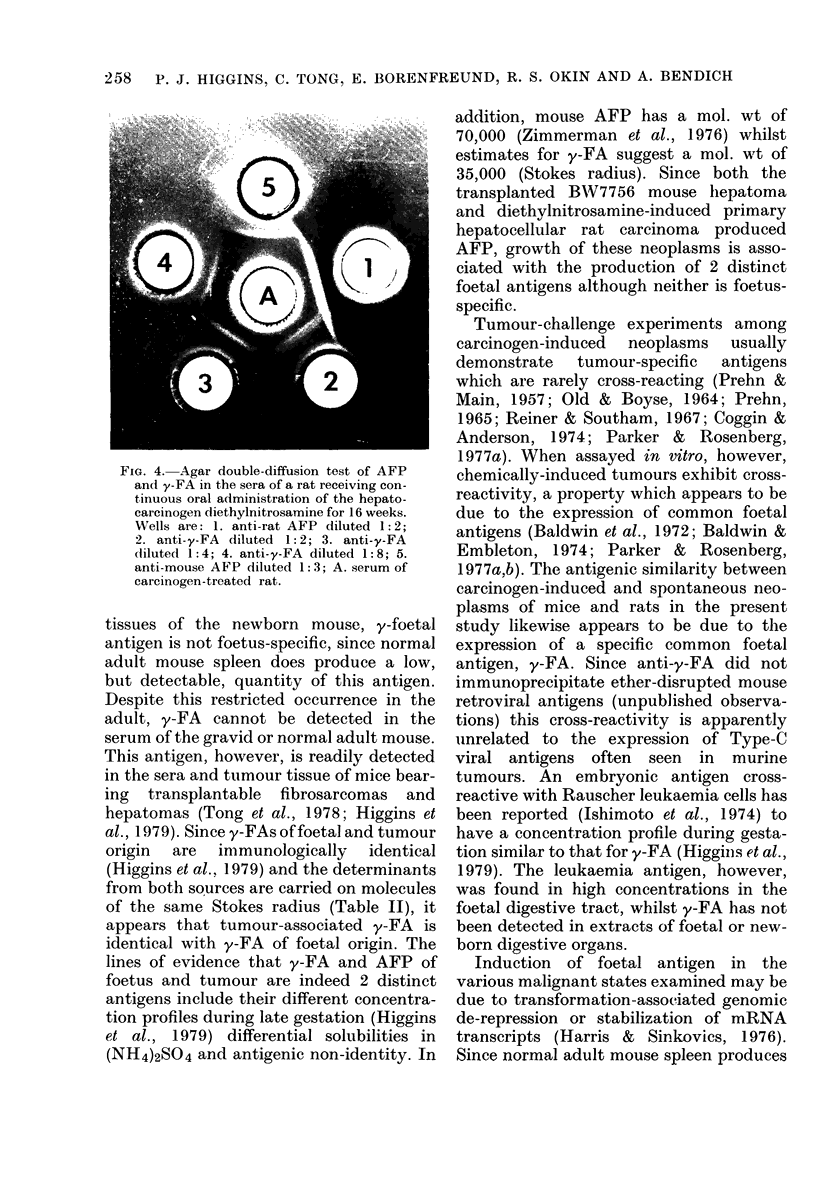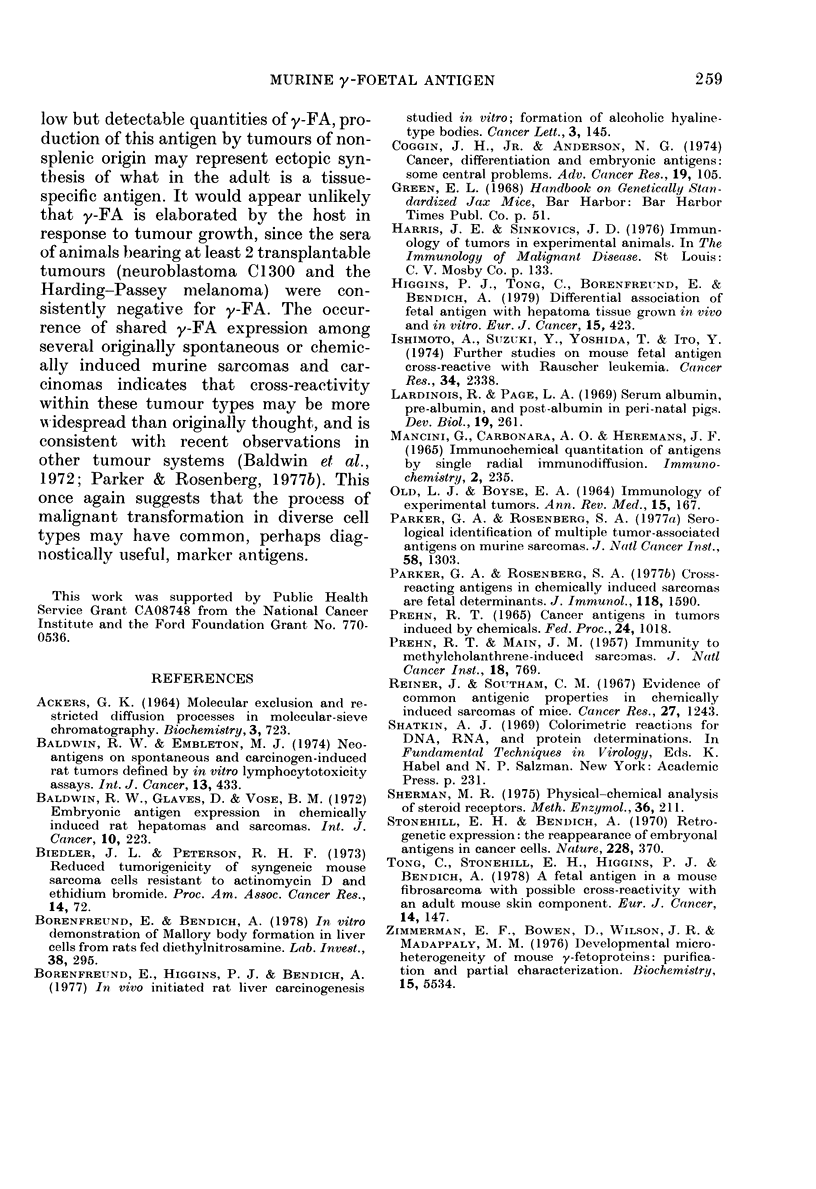# Tissue distribution and biochemical properties of an interspecific tumour-associated gamma foetal antigen.

**DOI:** 10.1038/bjc.1979.173

**Published:** 1979-08

**Authors:** P. J. Higgins, C. Tong, E. Borenfreund, R. S. Okin, A. Bendich

## Abstract

**Images:**


*Br. J. Cancer* (1979) **40**, 253

### TISSUE DISTRIBUTION AND BIOCHEMICAL PROPERTIES OF AN INTERSPECIFIC TUMOUR-ASSOCIATED GAMMA FOETAL ANTIGEN

P. J. HIGGINS,*‡ C. TONG,† E. BORENFREUND,* R. S. OKIN,* AND A. BENDICH*

*From the *Laboratory of Cell Biochemistry, Memorial Sloan–Kettering Cancer Center, New York, and the †Division of Experimental Pathology, Naylor Dana Institute for Disease Prevention, American Health Foundation, Valhalla, New York, U.S.A.*

Received 4 January 1979 Accepted 10 April 1979

**Summary.—A late-gestation neonatal antigen (gamma foetal antigen; $\gamma$-FA) immunologically and biochemically unrelated to murine $\alpha$-foetoprotein, was identified in several spontaneous and carcinogen-induced sarcomas and hepatic carcinomas of the mouse and rat. An approximate mol. wt of 35,000 for $\gamma$-FA from both foetus and tumour was obtained by molecular-sieve chromatography and sucrose-gradient centrifugation. Radial immunodiffusion analyses of organ extracts indicated that $\gamma$-FA could be found in several neonatal tissues, the highest concentration occurring in the spleen. In the 2-month-old mouse, only splenic tissue contained $\gamma$-FA and at much lower levels than in the organ of the newborn mouse.**

THE RE-EXPRESSION of certain common embryonic antigens has been observed in 2 hepatic tumours and a fibrosarcoma originally induced in rats with 4-dimethylaminoazobenzene and 3-methylcholanthrene respectively (Baldwin *et al.*, 1972). Recently, a late-gestation/neonatal antigen (gamma foetal antigen) was identified in the sera of mice bearing transplanted tumours derived from 3-methylcholanthrene-induced fibrosarcomas and a spontaneous hepatoma (Tong *et al.*, 1978; Higgins *et al.*, 1979). This expression of shared tumour-associated foetal antigens by neoplastic cells suggests that certain events associated with malignant transformation of diverse cell types may be similar. Analysis of the characterization and distribution of specific tumour-associated foetal antigens would, therefore, be essential to any comprehensive understanding of the antigenic composition of tumour cells.

In this paper, some of the biochemical properties of murine gamma foetal antigen ($\gamma$-FA) are described, as well as its tissue distribution in the foetal and adult mouse. Evidence is also presented for the occurrence of $\gamma$-FA in the sera of rats bearing primary carcinogen-induced hepatomas.

### MATERIALS AND METHODS

*Tumours and experimental animals.*—The BW7756 mouse hepatoma was obtained from the Jackson Laboratory (Bar Harbor, Maine) and maintained in s.c. animal passage in C57L/J mice. Mouse QUA fibrosarcoma was routinely passaged in C57BL/6 mice (Tong *et al.*, 1978). Female Wistar rats ($\sim$120 g) were allowed continuous access to drinking water containing 5 mg diethylnitrosamine/100 ml in order to induce primary hepatomas (Borenfreund *et al.*, 1977; Borenfreund & Bendich, 1978). The appearance of hepatocellular cancers in carcinogen-fed rats was monitored by agar double-diffusion test of sera, obtained by weekly tail bleedings, using rabbit antisera to rat $\alpha$-foetoprotein (AFP). Fresh tumour tissues and organs (0·2–5·0 g wet wt) obtained from neonatal and adult

‡ To whom reprint requests should be addressed.
Address for correspondence: Dr Paul J. Higgins, Laboratory of Cell Biochemistry, Memorial Sloan–Kettering Cancer Center, 1275 York Avenue, New York, New York 10021.

C57 mice were homogenized in 3 × the tissue weight of phosphate-buffered saline (PBS) using a Virtis 45 tissue homogenizer, and the 3000 *g* supernatant was collected (Stonehill & Bendich, 1970).

*Histology.*—Liver specimens from $AFP^+$ rats were fixed in Bouin's solution, embedded in paraffin and sectioned for histological examination. Tissues were stained with haematoxylin and eosin.

*Antisera.*—The production of an antiserum to murine γ-FA (anti-γ-FA) has been described (Tong *et al.*, 1978). Briefly, rabbits were immunized with PBS extracts of 3-methylcholanthrene-induced mouse QUA fibrosarcomas (Biedler & Peterson, 1973). The γ-globulin fraction of anti-QUA tumour serum was repeatedly absorbed by incubation at 37°C for 1 h with lyophilized extracts of normal adult internal organs and freshly collected adult mouse serum. After further incubation at 4°C for 5 h, the absorbed γ-globulin preparation was clarified by centrifugation at 12,000 *g*. The absorption procedure was continued until precipitin activity to adult mouse serum and all internal organs (except spleen) was removed, as determined by agar double-diffusion test. Immunoelectrophoretic analysis of extracts of QUA tumour and foetal mice with this absorbed antiserum disclosed a single precipitin arc of γ mobility in extracts of both foetus and tumour; agar double-diffusion test revealed the antigen (γ-FA) of tumour and foetus to be antigenically identical (Tong *et al.*, 1978; Higgins *et al.*, 1979).

Antiserum to rat AFP was obtained by methods similar to those previously described for preparation of anti-mouse AFP (Higgins *et al.*, 1979).

*Antigen quantitation.*—The relative amount of γ-FA in organ extracts of neonatal and adult C57BL/6 mice was determined by radial immunodiffusion assay (Mancini *et al.*, 1965; Tong *et al.*, 1978). For assay, 2·5 ml of 1% agarose (w/v) in Beckman B-2 buffer, pH 8·6, was mixed with 0·1 ml of anti-γ-FA and 0·1 ml of normal rabbit serum, and layered into empty Hyland Immuno-plate moulds (Hyland Laboratories, Costa Mesa, CA). Antigen wells 3·7 mm in diameter were cut and filled with 10 μl of organ extract of known protein concentration (Shatkin, 1969). Plates were incubated at 37°C and the diameters of the precipitin discs measured after 72 h. The radial immunodiffusion unit used for quantitation is defined as the square of the diameter (in mm) of the precipitin disc minus the square of the antigen well diameter per μg of protein assayed (Tong *et al.*, 1978).

*Sucrose-gradient sedimentation.*—Sedimentation of 0·5ml samples of BW7756 mouse hepatoma and QUA tumour extracts through continuous 5-20% sucrose/PBS gradients was done for 18 h at 45,000 rev/min in an SW 50·1 rotor, using a Beckman L-2 preparative ultracentrifuge. After centrifugation, the tubes were punctured from the bottom, 4-drop fractions collected and the position of γ-FA located by radial immunodiffusion. Marker proteins bovine serum albumin (4·6S, 67,000 mol. wt), ovalbumin (3·6S, 43,500 mol. wt) and lysozyme (2·1S, 17,200 mol. wt) were located in parallel gradients by UV absorption.

*Molecular-sieve chromatography.*—PBS extracts of Day 17 C57BL/6 foetal mice and QUA tumour were chromatographed on Sephadex G-200 using a K15/30 column (Pharmacia Fine Chem. Co., Piscataway, N.J.) which had been calibrated with proteins of known Stokes radii. Elution profiles were monitored with a Gilford Multisample Absorbance recorder at 280 nm. The peak of γ-FA elution was determined by radial immunodiffusion assay of 10μl aliquots from each fraction. The Stokes radius of γ-FA derived from tumour and embryo was calculated according to Ackers (1964) using data obtained from duplicate analyses.

*Solubility and heat-denaturation determinations.*—The solubility of hepatoma-extracted γ-FA was examined in Tris buffer and Tris buffer containing ammonium sulphate. Heat stability of γ-FA antigenic reactivity was determined in $10^{-3}$M Tris, pH 7·2.

*Double-diffusion assay.*—Agar double-diffusion assay of sera and tumour extracts used Hyland Immuno-plates, pattern "D". Wells were filled with 10 μl of antisera or test antigen solution, the plates sealed, and kept at 37°C for 72 h to allow the precipitin reaction to go to completion. When necessary, the precipitin patterns were stained with Amidoschwarz 10B (Lardinois & Page, 1969).

### RESULTS

### *Tissue distribution of γ-FA in organ extracts of neonatal and adult mice*

Determinations of relative γ-FA levels in organ extracts of neonatal mice revealed an equal distribution of antigen, per μg of extractable protein, in Day 1 newborn liver and lung (Table I). Slightly higher concentrations occurred in thymus, whilst the greatest concentration was seen in neonatal spleen. The only young adult mouse internal organ from which antigenic activity to anti-γ-FA could be extracted was the spleen. The 0·73 radial immunodiffusion units for splenic tissue from a 2-month-old mouse, however, was considerably less than the estimated 198·0 units for the same newborn mouse organ.

TABLE I.—*Quantitation of γ-foetal antigen in phosphate-buffered saline extracts* of mouse internal organs by single radial-immunodiffusion assay*

| | Radial immunodiffusion units† | |
|---|---|---|
| Tissue extracted | Neo-natal organs | Adult organs‡ |
| Liver | 9·8 ± 1·2 | 0 |
| Lung | 11·3 ± 1·4 | 0 |
| Thymus | 25·1 ± 3·2 | N.T. |
| Spleen | 197·8 ± 15·9 | 0·73 ± 0·01 |
| Brain | 0 | 0 |
| Kidney | 0 | 0 |
| Intestines | 0 | 0 |
| Heart | N.T. | 0 |

* Extracts prepared according to Stonehill & Bendich (1970).

† As described in "Methods"; data represent average of 8–10 individual assays.

‡ From 2-month-old mice.

N.T. not tested.

*Biochemical and biophysical properties of γ-FA*

Extraction of mouse BW7756 hepatoma tissue in $10^{-3}$M Tris buffer, pH 7·2, and centrifugation at 10,000 *g* produced a supernatant fraction containing γ-FA and AFP; the 2 antigens proved to be immunologically distinct (Fig. 1). Heating of this extract to 56°C for 30 min caused an 82% loss of soluble protein; agar double-diffusion analysis of the 10,000 *g* 56°C supernatant brought to 22°C showed that the antigenic reactivity of both AFP and γ-FA with their respective antisera was retained after heating. Dialysis of the 56°C-supernatant fraction of mouse hepatoma extract against 75% saturated $(NH_4)_2SO_4$ removed all immunoprecipitable AFP from solution, but all antigenic activity of γ-FA remained in the 75% saturated $(NH_4)_2SO_4$ supernatant. Sucrose-gradient centrifugation of 0·5 ml aliquots of mouse QUA or BW7756 tumour

FIG. 1.—Agar double-diffusion demonstration of antigenic non-identity of γ-FA and AFP. Wells are as follows: A, Saline extract of BW7756 mouse hepatoma; 1, anti-γ-FA diluted 1:8; 2, anti-γ-FA diluted 1:4; 3, anti-AFP. The precipitin line which formed with each of these antisera with their respective antigens was one of non-identity, indicating that AFP and γ-FA are distinct antigenic species.

FIG. 2.—Composite sucrose-sedimentation profiles of bovine serum albumin (BSA), ovalbumin, lysozyme and γ-FA. Position of reference proteins in the gradients were determined by UV absorption; γ-FA (from hepatoma and QUA tumour) was located by radial immunodiffusion. The antigen of QUA tumour and hepatoma was detected in Fractions 14 to 17. The precipitin-ring diameters for the QUA tumour fractions were: 14 (6·3 mm); 15 (8·0 mm); 16 (8·9 mm); 17 (7·6 mm). γ-FA from both QUA tumour and hepatoma extracts had a sedimentation coefficient of 3·5 S.

tissue PBS extracts and subsequent immunological monitoring of the fractionated gradients by radial-immunodiffusion assay indicated a sedimentation coefficient of 3·5 S for γ-FA (Fig. 2).

Sephadex G-200 chromatography of aliquots of QUA tumour and Day 17 mouse foetal-tissue extracts and radial-immunodiffusion assay of the eluted fractions for γ-FA yielded the elution profile of Fig. 3. It is evident from Fig. 3 and Table II that the position at which γ-FA eluted from G-200 Sephadex was almost identical for embryo and tumour. Dupli-

FIG. 3.—Sephadex G-200 chromatography of PBS extracts of mouse QUA tumour and Day 17 foetal mouse tissue. Elution profiles of tumour (closed circles) and foetal (open circles) extracts were monitored by absorption at 280 nm. Eluent fractions were assayed for γ-FA by radial immunodiffusion; open columns indicate the radial-immunodiffusion units in each fraction of foetal extract, and closed columns the units in tumour fractions. For Stokes-radius determination, the column was calibrated with reference proteins of known Stokes radii (Table II) and the γ-FA contribution to caeh fraction determined immunologically.

TABLE II.—*Computation of the Stokes radius of γ-FA according to Ackers* (1964)

| | $V_e$ (ml) | $V_e - V_o$ (ml) | $K_D = \frac{V_e - V_o}{V_i}$ | $a/r$ | $a$ (nm) | $r$ (nm) |
|---|---|---|---|---|---|---|
| Mouse gamma-globulin | 21·0 | 4·6 | 0·1783 | 0·3226 | 5·22 | 16·2 |
| Ovalbumin | 31·0 | 14·6 | 0·5659 | 0·1256 | 2·73 | 21·7 |
| Myoglobin | 33·4 | 17·0 | 0·6589 | 0·0944 | 1·90 | 20·1 |
| Cytochrome C | 34·0 | 17·6 | 0·6822 | 0·0873 | 1·65 | 18·9 |
| Bovine serum albumin | 26·4 | 10·0 | 0·3876 | 0·1971 | 3·70 | 18·8 |
| | | | | | Average | 19·2 ± 2·0 |
| QUA tumour extract I | 31·1 | 14·7 | 0·5698 | 0·1242 | 2·39 | |
| QUA tumour extract II | 30·5 | 14·1 | 0·5465 | 0·1325 | 2·54 | |
| | | | | Average | 2·47 | |
| 17-day embryo extract I | 30·9 | 14·5 | 0·5620 | 0·1269 | 2·44 | |
| 17-day embryo extract II | 31·5 | 15·1 | 0·5853 | 0·1188 | 2·28 | |
| | | | | Average | 2·36 | |

Estimated $a$ of murine γ-FA = 2·41 ± 0·11 μm

$V_e$: elution volume. Elution peak for protein standards was located by UV absorption; elution peak for γ-FA in tumour and embryo extracts was ascertained by radial immunodiffusion (see Fig. 3).

$V_o$: void volume as determined with blue dextran. Average $V_o$ of 4 separate determinations was 16·4 ml.

$K_D = \frac{V_e - V_o}{V_i}$: distribution coefficient (Ackers, 1964).

$V_i$: internal volume, obtained by determination of $V_{e\ phenylalanine} - V_o$ (*i.e.* 42·2 − 16·4 = 25·8 ml).

$a$: Stokes radius; the known values for gamma-globulin, ovalbumin, myoglobin, cytochrome C, and bovine serum albumin were as reported (Ackers, 1964) or as calculated from the diffusion coefficient (Sherman, 1975).

$r$: effective gel-pore radius (Ackers, 1964).

TABLE III.—*Distribution of γ-foetal antigen among tumour-bearing and control animals**

| Specimen | Species | Aetiology | Serum γ-FA | Tumour γ-FA |
|---|---|---|---|---|
| *Tumours* | | | | |
| QUA fibrosarcoma | Mouse | MCA†-induced: transplanted | + | + |
| BW7756 hepatoma | Mouse | Spontaneous: transplanted | + | + |
| Sarcoma 180‡ | Mouse | Spontaneous: transplanted | + | + |
| Metu fibrosarcoma§ | Mouse | MCA-induced: transplanted | + | + |
| Neuroblastoma 1300‡ | Mouse | Spontaneous: transplanted | − | − |
| Harding-Passey melanoma‡ | Mouse | Spontaneous: transplanted | − | − |
| Hepatocellular carcinoma | Rat | DENA‖-induced: primary | + | + |
| *Serum controls* | | | | |
| Normal adults | Mouse | | − | |
| Gravid females | Mouse | | − | |
| Multiparous females | Mouse | | − | |
| Normal adults | Rat | | − | |
| Gravid females | Rat | | − | |

* Agar double-diffusion assay.

† 3-methylcholanthrene.

‡ Green, 1968.

§ Tumour line established in this laboratory by inoculation of 0·1 ml of a 5 mg/ml solution of MCA in sesame oil into the hind leg of a C57BL/6 mouse. A transplantable fibrosarcoma developed 7 months later at the site of inoculation.

‖ Diethylnitrosamine.

cate analyses of γ-FA from both embryo and QUA tumour showed that the antigen from either source had about the same Stokes radius (Table II). The results of these various Stokes radii determinations suggest a mol. wt for γ-FA of ~35,000. Agar double-diffusion test of tumour extracts or sera from tumour-bearing mice revealed the presence of γ-FA in 4/6 neoplasms examined (Table III). This antigen was not detected in the sera of pregnant or multiparous mice. The biophysical and biochemical properties of γ-FA are summarized in Table IV.

To ascertain whether an antigen reactive with anti-mouse γ-FA could be induced in another species during the course of hepatocarcinogenesis, rats were fed diethylnitrosamine (50 parts/$10^6$ in the drinking water) to produce primary hepatocellular cancers. One rat out of 4, after 16 weeks of continuous carcinogen administration, developed elevated levels of AFP detectable by agar double-diffusion analysis of serum obtained by a test tail bleeding (Fig. 4). The serum of this animal was also positive for γ-FA. At necropsy the liver was found to contain numerous pea-sized nodules, and histological examination revealed hepatocellular carcinoma. Maintenance of this tumour by s.c. implantation into nude mice consistently yielded γ-$FA^+$ carcinomas.

TABLE IV.—*Properties of tumour-associated γ-FA*

Soluble in $10^{-3}$M Tris buffer, pH 7·2*

Antigenic activity stable to heating at 56°C, 30 min.

Soluble in 75% saturated $(NH_4)_2SO_4$

Gamma electrophoretic mobility†

Sedimentation coefficient of 3·5S

Stokes radius of 2·41 nm‡

Mol. wt ~35,000‡

* Agar double-diffusion test using anti-γ-FA serum diluted 1:40 and 1:80 with normal adult mouse serum.

† Tong *et al.*, 1978.

‡ As estimated by gel chromatography.

### DISCUSSION

The present results indicate that γ-FA occurs in several mouse sarcomas and a transplantable hepatic carcinoma. Moreover, a cross-reacting antigen was observed in the sera and tumour tissue of a rat bearing a diethylnitrosamine-induced hepatocellular carcinoma. Although present in apparently high yield in certain

FIG. 4.—Agar double-diffusion test of AFP and $\gamma$-FA in the sera of a rat receiving continuous oral administration of the hepatocarcinogen diethylnitrosamine for 16 weeks. Wells are: 1. anti-rat AFP diluted 1:2; 2. anti-$\gamma$-FA diluted 1:2; 3. anti-$\gamma$-FA diluted 1:4; 4. anti-$\gamma$-FA diluted 1:8; 5. anti-mouse AFP diluted 1:3; A. serum of carcinogen-treated rat.

tissues of the newborn mouse, $\gamma$-foetal antigen is not foetus-specific, since normal adult mouse spleen does produce a low, but detectable, quantity of this antigen. Despite this restricted occurrence in the adult, $\gamma$-FA cannot be detected in the serum of the gravid or normal adult mouse. This antigen, however, is readily detected in the sera and tumour tissue of mice bearing transplantable fibrosarcomas and hepatomas (Tong *et al.*, 1978; Higgins *et al.*, 1979). Since $\gamma$-FAs of foetal and tumour origin are immunologically identical (Higgins *et al.*, 1979) and the determinants from both sources are carried on molecules of the same Stokes radius (Table II), it appears that tumour-associated $\gamma$-FA is identical with $\gamma$-FA of foetal origin. The lines of evidence that $\gamma$-FA and AFP of foetus and tumour are indeed 2 distinct antigens include their different concentration profiles during late gestation (Higgins *et al.*, 1979) differential solubilities in $(NH_4)_2SO_4$ and antigenic non-identity. In addition, mouse AFP has a mol. wt of 70,000 (Zimmerman *et al.*, 1976) whilst estimates for $\gamma$-FA suggest a mol. wt of 35,000 (Stokes radius). Since both the transplanted BW7756 mouse hepatoma and diethylnitrosamine-induced primary hepatocellular rat carcinoma produced AFP, growth of these neoplasms is associated with the production of 2 distinct foetal antigens although neither is foetus-specific.

Tumour-challenge experiments among carcinogen-induced neoplasms usually demonstrate tumour-specific antigens which are rarely cross-reacting (Prehn & Main, 1957; Old & Boyse, 1964; Prehn, 1965; Reiner & Southam, 1967; Coggin & Anderson, 1974; Parker & Rosenberg, 1977*a*). When assayed *in vitro*, however, chemically-induced tumours exhibit cross-reactivity, a property which appears to be due to the expression of common foetal antigens (Baldwin *et al.*, 1972; Baldwin & Embleton, 1974; Parker & Rosenberg, 1977*a,b*). The antigenic similarity between carcinogen-induced and spontaneous neoplasms of mice and rats in the present study likewise appears to be due to the expression of a specific common foetal antigen, $\gamma$-FA. Since anti-$\gamma$-FA did not immunoprecipitate ether-disrupted mouse retroviral antigens (unpublished observations) this cross-reactivity is apparently unrelated to the expression of Type-C viral antigens often seen in murine tumours. An embryonic antigen cross-reactive with Rauscher leukaemia cells has been reported (Ishimoto *et al.*, 1974) to have a concentration profile during gestation similar to that for $\gamma$-FA (Higgins *et al.*, 1979). The leukaemia antigen, however, was found in high concentrations in the foetal digestive tract, whilst $\gamma$-FA has not been detected in extracts of foetal or newborn digestive organs.

Induction of foetal antigen in the various malignant states examined may be due to transformation-associated genomic de-repression or stabilization of mRNA transcripts (Harris & Sinkovics, 1976). Since normal adult mouse spleen produces low but detectable quantities of $\gamma$-FA, production of this antigen by tumours of non-splenic origin may represent ectopic synthesis of what in the adult is a tissue-specific antigen. It would appear unlikely that $\gamma$-FA is elaborated by the host in response to tumour growth, since the sera of animals bearing at least 2 transplantable tumours (neuroblastoma C1300 and the Harding–Passey melanoma) were consistently negative for $\gamma$-FA. The occurrence of shared $\gamma$-FA expression among several originally spontaneous or chemically induced murine sarcomas and carcinomas indicates that cross-reactivity within these tumour types may be more widespread than originally thought, and is consistent with recent observations in other tumour systems (Baldwin *et al.*, 1972; Parker & Rosenberg, 1977*b*). This once again suggests that the process of malignant transformation in diverse cell types may have common, perhaps diagnostically useful, marker antigens.

This work was supported by Public Health Service Grant CA08748 from the National Cancer Institute and the Ford Foundation Grant No. 770-0536.

### REFERENCES

ACKERS, G. K. (1964) Molecular exclusion and restricted diffusion processes in molecular-sieve chromatography. *Biochemistry*, **3**, 723.

BALDWIN, R. W. & EMBLETON, M. J. (1974) Neoantigens on spontaneous and carcinogen-induced rat tumors defined by *in vitro* lymphocytotoxicity assays. *Int. J. Cancer*, **13**, 433.

BALDWIN, R. W., GLAVES, D. & VOSE, B. M. (1972) Embryonic antigen expression in chemically induced rat hepatomas and sarcomas. *Int. J. Cancer*, **10**, 223.

BIEDLER, J. L. & PETERSON, R. H. F. (1973) Reduced tumorigenicity of syngeneic mouse sarcoma cells resistant to actinomycin D and ethidium bromide. *Proc. Am. Assoc. Cancer Res.*, **14**, 72.

BORENFREUND, E. & BENDICH, A. (1978) *In vitro* demonstration of Mallory body formation in liver cells from rats fed diethylnitrosamine. *Lab. Invest.*, **38**, 295.

BORENFREUND, E., HIGGINS, P. J. & BENDICH, A. (1977) *In vivo* initiated rat liver carcinogenesis studied *in vitro*; formation of alcoholic hyaline-type bodies. *Cancer Lett.*, **3**, 145.

COGGIN, J. H., JR. & ANDERSON, N. G. (1974) Cancer, differentiation and embryonic antigens: some central problems. *Adv. Cancer Res.*, **19**, 105.

GREEN, E. L. (1968) *Handbook on Genetically Standardized Jax Mice*, Bar Harbor: Bar Harbor Times Publ. Co. p. 51.

HARRIS, J. E. & SINKOVICS, J. D. (1976) Immunology of tumors in experimental animals. In *The Immunology of Malignant Disease.* St Louis: C. V. Mosby Co. p. 133.

HIGGINS, P. J., TONG, C., BORENFREUND, E. & BENDICH, A. (1979) Differential association of fetal antigen with hepatoma tissue grown *in vivo* and *in vitro*. *Eur. J. Cancer*, **15**, 423.

ISHIMOTO, A., SUZUKI, Y., YOSHIDA, T. & ITO, Y. (1974) Further studies on mouse fetal antigen cross-reactive with Rauscher leukemia. *Cancer Res.*, **34**, 2338.

LARDINOIS, R. & PAGE, L. A. (1969) Serum albumin, pre-albumin, and post-albumin in peri-natal pigs. *Dev. Biol.*, **19**, 261.

MANCINI, G., CARBONARA, A. O. & HEREMANS, J. F. (1965) Immunochemical quantitation of antigens by single radial immunodiffusion. *Immunochemistry*, **2**, 235.

OLD, L. J. & BOYSE, E. A. (1964) Immunology of experimental tumors. *Ann. Rev. Med.*, **15**, 167.

PARKER, G. A. & ROSENBERG, S. A. (1977*a*) Serological identification of multiple tumor-associated antigens on murine sarcomas. *J. Natl Cancer Inst.*, **58**, 1303.

PARKER, G. A. & ROSENBERG, S. A. (1977*b*) Cross-reacting antigens in chemically induced sarcomas are fetal determinants. *J. Immunol.*, **118**, 1590.

PREHN, R. T. (1965) Cancer antigens in tumors induced by chemicals. *Fed. Proc.*, **24**, 1018.

PREHN, R. T. & MAIN, J. M. (1957) Immunity to methylcholanthrene-induced sarcomas. *J. Natl Cancer Inst.*, **18**, 769.

REINER, J. & SOUTHAM, C. M. (1967) Evidence of common antigenic properties in chemically induced sarcomas of mice. *Cancer Res.*, **27**, 1243.

SHATKIN, A. J. (1969) Colorimetric reactions for DNA, RNA, and protein determinations. In *Fundamental Techniques in Virology*, Eds. K. Habel and N. P. Salzman. New York: Academic Press. p. 231.

SHERMAN, M. R. (1975) Physical–chemical analysis of steroid receptors. *Meth. Enzymol.*, **36**, 211.

STONEHILL, E. H. & BENDICH, A. (1970) Retrogenetic expression: the reappearance of embryonal antigens in cancer cells. *Nature*, **228**, 370.

TONG, C., STONEHILL, E. H., HIGGINS, P. J. & BENDICH, A. (1978) A fetal antigen in a mouse fibrosarcoma with possible cross-reactivity with an adult mouse skin component. *Eur. J. Cancer*, **14**, 147.

ZIMMERMAN, E. F., BOWEN, D., WILSON, J. R. & MADAPPALY, M. M. (1976) Developmental microheterogeneity of mouse $\gamma$-fetoproteins: purification and partial characterization. *Biochemistry*, **15**, 5534.